# Non-SMC condensin I complex proteins control chromosome segregation and survival of proliferating cells in the zebrafish neural retina

**DOI:** 10.1186/1471-213X-9-40

**Published:** 2009-07-08

**Authors:** Sabine Seipold, Florian C Priller, Paul Goldsmith, William A Harris, Herwig Baier, Salim Abdelilah-Seyfried

**Affiliations:** 1 Max Delbrück Center (MDC) for Molecular Medicine, Berlin, Robert-Rössle Str. 10, 13125 Berlin, Germany; 2 Department of Anatomy, Cambridge University, Cambridge, CB2 3DY, UK; 3 Department of Physiology, University of California, San Francisco, CA 94158-2722, USA; 4 Department of Neurology, Royal Victoria Infirmary, Queen Victoria Road, Newcastle upon Tyne, NE1 4LP, UK

## Abstract

**Background:**

The condensation of chromosomes and correct sister chromatid segregation during cell division is an essential feature of all proliferative cells. Structural maintenance of chromosomes (SMC) and non-SMC proteins form the condensin I complex and regulate chromosome condensation and segregation during mitosis. However, due to the lack of appropriate mutants, the function of the condensin I complex during vertebrate development has not been described.

**Results:**

Here, we report the positional cloning and detailed characterization of retinal phenotypes of a zebrafish mutation at the *cap-g *locus. High resolution live imaging reveals that the progression of mitosis between prometa- to telophase is delayed and that sister chromatid segregation is impaired upon loss of CAP-G. CAP-G associates with chromosomes between prometa- and telophase of the cell cycle. Loss of the interaction partners CAP-H and CAP-D2 causes cytoplasmic mislocalization of CAP-G throughout mitosis. DNA content analysis reveals increased genomic imbalances upon loss of non-SMC condensin I subunits. Within the retina, loss of condensin I function causes increased rates of apoptosis among cells within the proliferative ciliary marginal zone (CMZ) whereas postmitotic retinal cells are viable. Inhibition of p53-mediated apoptosis partially rescues cell numbers in *cap-g *mutant retinae and allows normal layering of retinal cell types without alleviating their aberrant nuclear sizes.

**Conclusion:**

Our findings indicate that the condensin I complex is particularly important within rapidly amplifying progenitor cell populations to ensure faithful chromosome segregation. In contrast, differentiation of postmitotic retinal cells is not impaired upon polyploidization.

## Background

SMC family proteins [1,2] are essential regulators of chromosomal organization in mitotic and meiotic cell cycles and control sister chromatid cohesion and separation, mitotic condensation, recombinational repair, and chromosome-wide gene regulation [3,4]. Two SMC proteins, SMC2 and SMC4, heterodimerize to form an active ATPase at the core of condensin I and condensin II protein complexes that are essential for the condensation and stability of chromosomes during mitosis in eukaryotes ranging from yeast to humans [5-8]. In addition to the SMC2/SMC4 core proteins, the condensin I complex contains the kleisin subunit CAP-H and the two HEAT domain proteins CAP-D2 and CAP-G. The non-SMC subunits of the condensin complexes have been proposed to activate DNA supercoiling and looping activity of the SMC-ATPases and to play essential roles in directing the association of the condensin holocomplex onto chromosomes at the correct mitotic stage [4,6]. Components of the condensin I complex are cytoplasmic during interphase and are targeted to chromatin after the breakdown of the nuclear membrane during prometaphase through the A kinase-anchoring protein AKAP95 [9,10].

In budding and fission yeasts as well as in *Xenopus laevis *egg extracts, condensin has an important chromosome condensation activity [11-13]. In budding yeast, loss of any component of the condensin complex causes chromosome condensation and segregation defects [2,13-18]. Similarly, in HeLa cells, depletion of either condensin I or condensin II subunits causes defective chromosome condensation. This effect is enhanced upon simultaneous depletion of subunits from both complexes [19]. In *C. elegans *and *Drosophila*, loss of condensin subunits results in the formation of chromosome bridges due to the failure of sister chromatids to separate completely during anaphase [20-23]. In contrast to sister chromatid segregation, the compaction of chromosomes in metazoan organisms is not entirely dependent on condensin complexes. Genetic analyses of different SMC and non-SMC subunits in several metazoan organisms have demonstrated that chromosomal compaction occurs in the absence of condensin [21,23-30].

In addition to their roles in mitosis, condensin complexes have been shown to regulate transcriptional expression by modulating heterochromatin function during interphase [16,21,22,31-33]. In comparison, the *in vivo *analysis of condensin function in vertebrates has been scarce. Overexpression of More than blood (MTB), the murine homolog of the condensin II subunit CAP-G2, in murine erythroleukemia cells promotes their erythroid differentiation [34]. A mutation at the murine condensin II *kleisin β *locus (*cap-h2*) disrupts T-cell differentiation [35]. To date, vertebrate mutants of condensin I complex components have not been described.

In this study, we report the positional cloning and detailed phenotypic characterization of the zebrafish *cap-g *mutation. Functional analysis of CAP-G and of its interaction partners CAP-H and CAP-D2 reveals that the condensin I complex ensures the correct segregation of chromosomes during mitosis and maintains the diploid state. Within the retina, proliferative cells in the ciliary marginal zone (CMZ) are particularly sensitive to the loss of the condensin I protein complex, resulting in increased apoptotic cell death whereas postmitotic cells differentiate and are viable. Survival and laminar organization of condensin I complex deficient retinal cells are partially restored upon inhibition of p53-mediated apoptotic cell death, whereas abnormal ploidy levels remain unchanged. These findings imply that differentiation of retinal cells is not impaired upon polyploidization.

## Results

### The zebrafish *creature from the black lagoon (cbl) *locus encodes the condensin I protein CAP-G

**Figure 1:**
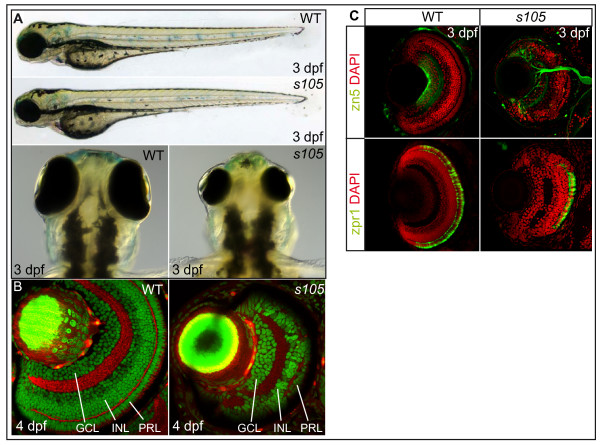
***s105 *mutant embryos have a strong reduction in retinal cell number**. (A) Wild-type (WT) and *s*105 mutant embryo at 3 dpf. The *s*105 mutant is characterized by small eyes. (B) Transverse vibratome sections through embryonic retinae counterstained with phalloidin to visualize plexiform layers (red) and propidium iodide to mark nuclei (green). In *s*105 mutants, the retina is smaller but some stratification of the retinal layers is present. (C) Transverse vibratome sections through embryonic retinae counterstained with DAPI to visualize nuclei (red), with the zn5 antibody to detect ganglion cells and the optic nerve (green), or with the zpr1 antibody to detect red/green double cones (green). GCL, ganglion cell layer; INL, inner nuclear layer; PRL, photoreceptor cell layer.

The *cbl*^*s*105 ^mutant allele was isolated in a screen for retinal and behavioral mutants [36,37]. Whereas trunk and tail regions are of normal size, *cbl*^*s*105 ^mutants show a severe size reduction of the retina and head compared with wild-type embryos (Figure 1A). We first focused our attention on retinal organization between 3–5 days post fertilization (dpf). Due to the great temporal precision with which the different cell layers are formed during development and due to the availability of cell type specific markers, we could efficiently analyze the organization of this tissue [38]. Among the three major cell-body layers of which the retina is composed of, the innermost layer consists of ganglion cells (GCL) which are the earliest retinal cell population to differentiate (Figure 1B). The intermediate nuclear layer (INL) consists of amacrine, bipolar and horizontal cells. Finally, the outermost layer contains the photoreceptor cells (PRL) which are the latest differentiating neurons within the retina. In *cbl*^*s*105 ^mutant retinae, the typical organization and specification of several major retinal cell types and overall retinal patterning was apparently preserved, as assessed using two specific markers for differentiated neurons (Figure 1B, C). Strikingly, the number of retinal cells within each of the three layers was severely reduced by more than half (see below). This finding suggested a role of the *cbl *locus in proliferation or survival of all retinal cell types.

**Figure 2:**
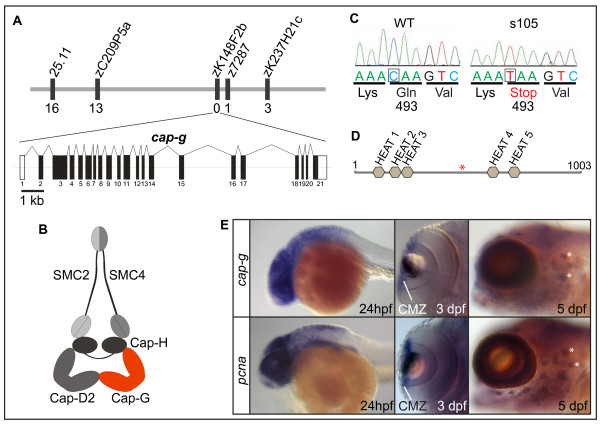
**The zebrafish *cap-g *gene is mutated in *s105***. (A) Representation of the genetic map of the *cap-g *locus on linkage group 1 (LG1) and exon/intron structure of the *cap-g *transcript. Some of the markers utilized for cloning the mutation are indicated and the number of recombinants among 2982 meioses is indicated below each marker. (B) Schematic model of the associated condensin I complex. (C) Comparison of sequence data for wild-type and *s*105 mutant alleles. The *s*105 mutation generates a premature stop codon. (D) Schematic diagram of the Cap-G protein which contains several predicted HEAT domains. The *s*105 mutation generates a premature stop codon (red asterisk) that truncates more than half of the protein. (E) Comparison of *cap-g *expression with that of *pcna *by whole-mount *in situ *hybridization. Overlapping expression with *pcna *within the brain, the CMZ of the retina, which contains retinal stem cells, and within neuromasts of the lateral line organ (white asterisks) indicates that *cap-g *is required within proliferative cells.

Using a positional cloning strategy, we mapped *cbl *to linkage group 1. Fine mapping of the locus with an F2 mapping panel using a simple-sequence-polymorphism-marker-based map, revealed that the mutation was positioned within a critical interval defined by markers z7287 (1/2982 recombinants) and 25.11 (16/2982 recombinants). By alignment of genes within the genomic region, we found synteny with Tetraodon and human genomic sequences which led to the prediction that *slit2 *should be present within the interval. This resulted in the identification of bacterial artificial chromosome (BAC) zK148F2 and allowed the assembly of overlapping BACs that covered the entire unknown interval (ending with zC209P5a). Subsequently, we failed to detect any recombinants out of 2982 meioses for a marker that was based on a BAC within this interval (zK148F2b) (Figure 2A). Analysis of this BAC using Genscan  did not reveal the presence of any open reading frames (ORFs), but five ORFs were predicted within the overlapping BAC clone zK215B13. Among these was an ORF encoding a protein with high similarity to human and other eukaryotic condensin I complex subunit CAP-G orthologs, the function of which was well in line with an involvement in cell proliferation (Figure 2B) [see Additional file 1]. Sequencing of the genomic *cbl*^*s*105 ^locus revealed a C-to-T base change which creates a premature stop codon causing a predicted truncation of the CAP-G protein at position 493 (Figure 2C, D). This finding was confirmed by sequencing of full-length cDNA from mutant and wild-type sibling embryos. Thus, the mutation in the *cbl*^*s*105 ^locus results in a protein that lacks more than 50% of the normal protein, including several predicted HEAT domains and, therefore, most likely results in a complete loss of function. *In situ *hybridizations showed that between 3–5 dpf, *cap-g *is strongly expressed within the neural retina and CNS in a pattern that is overlapping with *proliferative cell nuclear antigen *(*pcna*), a gene expressed within proliferative cells (Figure 2E).

The presence of maternally derived mRNA and probably also protein of condensin I complex components could mask their earliest developmental functions during blastula and gastrula stages [see Additional file 2]. It has been shown that maternal cell cycle gene deposits can last over a week especially in slowly dividing cells of the trunk and tail region [39]. To further verify that the *cbl *locus encodes CAP-G, we generated an ATG-directed antisense oligonucleotide morpholino (MO) that caused the same mitotic phenotypes as observed in *cbl*^*s*105 ^mutants (see below)[40]. However, consistent with an interference of this MO with maternal *cap-g *mRNA translation, *cap-g *morphant embryos displayed mitotic defects already during gastrula stages which preceded those of *cbl*^*s*105 ^mutants that occurred after somitogenesis stages. Moreover, *cap-g *morphants died during early somitogenesis (wild-type, n = 0/52 embryos dead at 24 hpf; *cap-g *morphants, n = 41/49 embryos dead at 24 hpf) [see Additional file 3]. In summary, these results establish that *cbl*^*s*105 ^is a severe mutant allele at the zebrafish *cap-g *locus, which we subsequently refer to as *cap-g*^*s*105^. Moreover, maternally provided *cap-g *mRNA stores persist at least through early embryogenesis and are sufficient to ensure development through gastrulation/early segmentation stages.

### Loss of CAP-G causes a severe reduction in retinal cell numbers, which is partly caused by p53-mediated apoptosis

**Figure 3:**
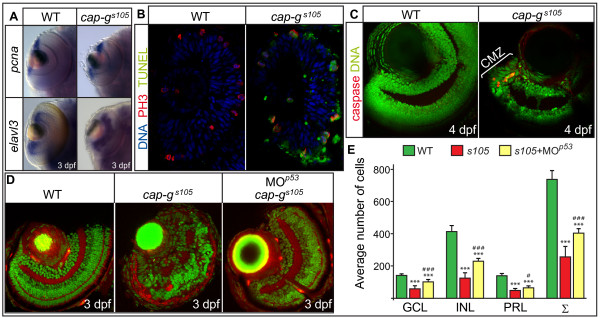
**Loss of CAP-G causes p53-mediated apoptosis within the retina**. (A) Retinal expression of the proliferation marker *pcna *or of neurogenesis marker *elval3 *is not affected in *cap-g*^*s*105 ^mutants as detected by whole-mount *in situ *hybridizations at 3 dpf. (B) Transverse cryosections of embryonic retinae were stained against phosphorylated histone 3 which marks mitotic nuclei (red), TUNEL to detect apoptotic cells (green), and nuclei counterstained with DAPI (blue) at 24 hpf. Predominantly mitotic cells which divide at the ventricular side of the retina are apoptotic in *cap-g*^*s*105 ^mutants at 24 hpf. (C) Transverse vibratome sections of 4 dpf retinae stained against activated caspase 3 to detect apoptotic cells (red) and nuclei counterstained with propidium iodide (green). At this stage, proliferation is restricted to the CMZ. In *cap-g*^*s*105 ^mutants, cell death is restricted to the CMZ which indicates that proliferative cells are eliminated. (D) Transverse vibratome sections of embryonic retinae counterstained with phalloidin to visualize plexiform layers (red) and propidium iodide (green). *cap-g*^*s*105 ^mutants injected with MO^*p*53^show a rescue of retinal development and display correct retinal layering. (E) Quantification of cell numbers within different retinal cell layers. Propidium iodide stained transverse retinal sections were used to determine average counts for wild-type (n = 9 section planes, 5 embryos), *cap-g*^*s*105 ^mutants (n = 11 section planes, 7 embryos) or *cap-g*^*s*105 ^mutant/*p*53 morphants (n = 9 section planes, 6 embryos). The average sum of *cap-g*^*s*105 ^mutant retinal cells is reduced by 65% compared with wild-type. In comparison, the average sum of *cap-g*^*s*105 ^mutant/*p53* morphant retinal cells is reduced only by 43% compared with wild-type. Therefore, the severe reduction in retinal cell numbers is in part caused by p53-mediated apoptosis. Data represent average cell numbers per retina ± SD. T-test p-values for cell number differences in comparison to wild-type: ***, p < 0.001; and in comparison to *cap-g*^*s*105^: #, p < 0.05; ### p < 0.001.

The neural retina is a tissue which is largely derived from a small pool of highly proliferative progenitor cells that are located within the proliferative zone of the retina, the CMZ [41]. To characterize the expression of genes involved in proliferation and neurogenesis within *cap-g*^*s*105 ^mutants, we performed *in situ *hybridizations. At 3 dpf, *pcna *is expressed exclusively within the CMZ (Figure 3A). Similar to the expression in wild-type, *pcna *was correctly expressed in the CMZ of *cap-g*^*s*105 ^mutants. Expression of *elavl3*, which is a marker of differentiated ganglion cells of the innermost retinal layer and of amacrine cells in the INL at 3–5 dpf [42], was not affected in *cap-g*^*s*105 ^mutants. These results indicated that the pattern of neurogenesis is not altered upon loss of CAP-G. Moreover, the presence of axonal tracts indicated that development is not generally delayed in *cap-g*^*s*105 ^mutants. Therefore, loss of retinal cell number in *cap-g*^*s*105 ^mutants is likely not caused by transcriptional silencing of genes involved in proliferation or differentiation as assessed by several markers (*pcna*, *elavl3*, zpr-1, zn5, zrf-1).

The severe reduction of eye and head regions in *cap-g*^*s*105 ^mutants led us to examine whether proliferation was affected. To this end, we stained mitotic cells with an antibody against phosphorylated histone H3 (PH3) to label mitotic nuclei. At 30 hours post fertilization (hpf), there was no significant difference in the number of mitotic cells throughout the entire embryo between wild-type (278 ± 43 mitotic cells) and mutants (229 ± 33 mitotic cells; p > 0.05). In contrast, apoptotic events were much more frequent in *cap-g*^*s*105 ^mutants compared to wild-type, as assayed by staining with acridine orange. Analysis of 24 hpf retinae revealed that many PH3-positive mitotic cells displayed TUNEL positive apoptotic nuclei (Figure 3B). At later stages, we detected cell death using an antibody against activated caspase 3 and found that apoptosis was largely restricted to the proliferative CMZ of the neural retina which suggests that proliferative retinal progenitor cells are primarily affected by loss of CAP-G, whereas postmitotic and terminally differentiated cells of the retina are viable (Figure 3C). This observation prompted us to test whether suppression of apoptotic cell death via inactivation of p53 would rescue the *cap-g*^*s*105 ^mutant phenotype [43]. Indeed, injection of 150 μM MO^*p*53 ^led to a significant increase in cell numbers throughout all retinal cell layers within *cap-g*^*s*105 ^embryos at 3 dpf, which were identified by PCR genotyping (Figure 3D, E). Only regions with a clear separation of the INL, GCL and PRL were assessed in this analysis. Taken together, these results demonstrate that CAP-G is required in proliferative rather than in postmitotic differentiated cells. Moreover, the severe reduction in retinal cell numbers is, in part, caused by p53-mediated apoptosis.

### Loss of CAP-G causes increased adhesion of sister chromatids during anaphase and aberrant nuclear sizes and shapes

**Figure 4:**
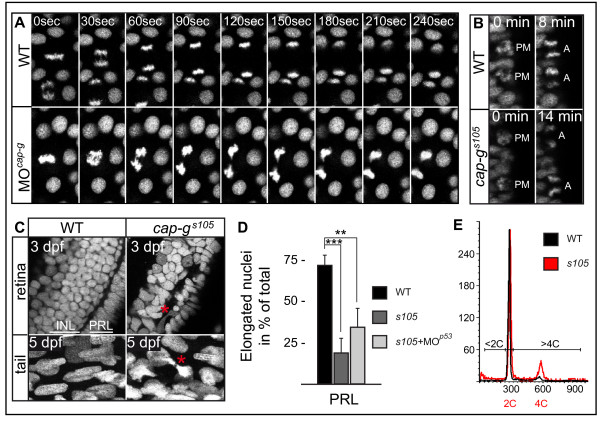
**Defective sister chromatid separation and aberrant nuclear sizes and shapes upon loss of Cap-G**. (A) Selected images from a timelapse recording of mitotic divisions at gastrula stages analyzed in *Tg [H2A::GFP] *transgenic embryos. *cap-g *morphant embryos display defective sister chromatid separation and abnormal chromatid morphology during anaphase. (B) Selected images from a timelapse analysis of mitotic divisions within the ventricular zone of the neural tube at 32 hpf. Progression from prometaphase, when condensed chromosomes are visible, to anaphase is significantly delayed in *cap-g*^*s*105 ^mutants. (C) Transverse vibratome sections through retinae counter-stained with propidium iodide reveal aberrant nuclear sizes and morphologies within *cap-g*^*s*105 ^mutants at 3 dpf. The strict retinal layering into inner INL and PRL is not recognizable. Red asterisk indicates decondensed nucleus with chromatid bridge/non-disjunction event. Similarly, the non-neural tail region of *cap-g*^*s*105 ^mutants contains cells with incomplete separation of chromatids (red asterisk). (D) In the wild-type retina, most nuclei in the photoreceptor layer have an elongated appearance. In *cap-g*^*s*105 ^mutant or *cap-g*^*s*105 ^mutant/*p*53 morphant retinae, most PRL nuclei fail to elongate. Data represent mean ± SD, n ≥ 115 for each retinal layer and genotype; **, p < 0.01; ***, p < 0.005. (E) Representative histograms from FACS analysis of propidium idodide stained nuclei suspensions. Whereas at 72 hpf most cells in the wild-type are diploid (2C; 1C = haploid genome equivalent), *cap-g*^*s*105 ^mutants harbor an increased fraction of cells with a genomic content >2C, especially in the tetraploid range (4C).

To elucidate the potential role of CAP-G in zebrafish chromosome condensation and mitosis, we employed a transgenic line of zebrafish which expresses a Histone2A::GFP fusion protein (*Tg *[*H2A::GFP*]) for high-resolution confocal live imaging of these processes during development [44]. Transgenic embryos were co-injected with MO^*cap-g *^and MO^*p*53 ^to avoid both specific and non-specific apoptosis caused by MO^*cap-g *^injection. During gastrula stages (between 70%-epiboly and tailbud stages), embryos were imaged within the animal cap region of the gastrula. Clearly, the dynamics of mitotic divisions was significantly perturbed upon loss of CAP-G (Figure 4A). Most strikingly, during ana- and telophase the distance of sister chromatids was strongly reduced in *cap-g *morphants and chromatids were abnormally shaped (Figure 4A).

To solidify our findings from morphants, we next tested whether similar mitotic defects could be observed in *cap-g*^*s*105 ^mutant embryos. To this end, we analyzed nuclear divisions by high-resolution live imaging at 32 hpf in *Tg *[*H2A::GFP*] transgenic embryos that were PCR genotyped (n = 3 *cap-g*^*s*105 ^mutant embryos analyzed). We focused on the ventricular highly proliferative zone of the CNS at the hindbrain level. Analysis of mitotic stages revealed that in wild-type embryos, the progression from prometa- to anaphase takes between 7–12 minutes (0% of mitoses delayed, n = 39 mitoses analyzed) (Figure 4B) [see Additional file 4]. In *cap-g*^*s*105 ^mutants, the progression from prometa- to anaphase was frequently delayed to 12–18 minutes (58.3% of mitoses delayed to >12 minutes, n = 24 mitoses analyzed) (Figure 4B) [see Additional file 5]. Taken together, live imaging demonstrates that the zebrafish CAP-G protein is an essential player in chromatid segregation during mitosis. Moreover, loss of CAP-G affects the timely progression of mitosis from prometa- to telophase.

### CAP-G is required for the maintenance of correct nuclear sizes and shapes

Since chromatid segregation is delayed during mitosis in CAP-G deficient embryos, we investigated nuclear shapes and sizes in *cap-g*^*s*105^ mutants. As expected for a failure of sister chromatids to completely segregate, we found that *cap-g*^*s*105 ^mutant retinal cells at 3 dpf contained many nuclei that appeared larger compared with those of wild-type and quantified those differences (Table 1). Moreover, many examples of non-disjunction events between neighboring nuclei were observed which provided further evidence for chromatid separation defects during mitosis (Figure 4C, red asterisks). Analysis of nuclei within the tail region also revealed the presence of chromosome segregation defects in *cap-g*^*s*105 ^mutants (Figure 4C). To quantify morphological differences between wild-type and *cap-g*^*s*105 ^mutant retinal nuclei, we performed nuclear circularity measurements. In the wild-type retina most nuclei in the PRL have an elongated appearance. In *cap-g*^*s*105 ^mutant retinae, significant deviations from wild-type nuclear shapes were detected in the PRL (Figure 4D), which in part is rescued by injection of MO^*p*53^. Together, these findings show that CAP-G is essential for the maintenance of correct nuclear sizes and shapes.

**Table 1:** Quantification of nuclear sizes in wild-type and *cap-g*^*s*105 ^retinal cross sections.

	nuclear cross section area			average nuclear	*t*-test	
	<20 μm^2^	20–40 μm^2^	>40 μm^2^	cross section area	vs. WT	n
WT	9.4%	87.9%	2.8%	17.3 ± 5.9 μm^2^		470
*cap-g*^*s*105^	15.9%	73.2%	10.9%	19.0 ± 10.8 μm^2^	p > 0.01	395
*cap-g*^*s*105 ^+ MO^*p*53^	13.2%	72.9%	13.9%	20.0 ± 9.9 μm^2^	p > 0.00001	409

Table showing the differences in nuclear cross section areas of nuclei from wild-type, *cap-g*^*s*105 ^mutant and *cap-g*^*s*105 ^mutant/*p53 *morphant embryos. Both *cap-g*^*s*105 ^mutant and *cap-g*^*s*105 ^mutant/*p53 *morphant retinae contain a significantly higher subset of smaller and larger nuclei compared to wild-type. Data represent mean ± SD.

### Loss of other non-SMC condensin I genes phenocopies *cap-g*^*s*105^

**Figure 5:**
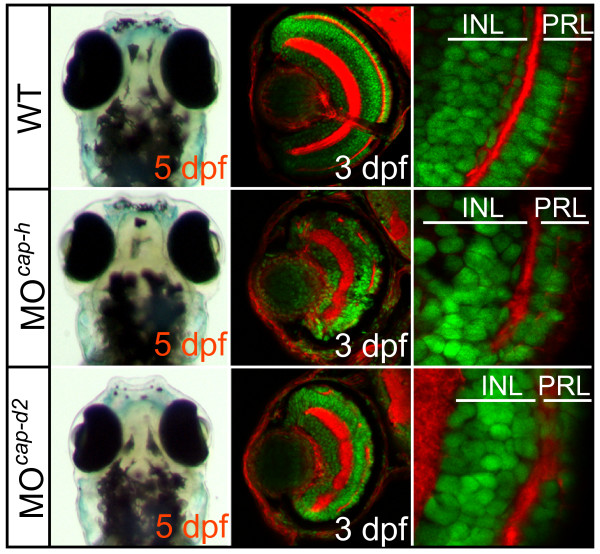
**CAP-H and CAP-D2 are essential components of the condensin I complex**. Morphant embryos show smaller heads and eyes. Transverse vibratome sections of embryonic retinae counterstained with phalloidin to visualize plexiform layers (red) and propidium iodide to mark nuclei (green). *cap-h *or *cap-d2 *morphant retinae are smaller in size. Similar to *cap-g*^*s*105^ mutant retinae, nuclei are aberrantly shaped and sized which is indicative of polyploidy/genomic imbalances. INL, inner nuclear layer; PRL, photoreceptor cell layer.

To assess the role of the non-SMC condensin I proteins CAP-D2 and CAP-H (Figure 2B) during zebrafish development, we identified the homologous genes from the zebrafish whole genome sequence and designed ATG directed MOs for functional studies. Injection of MO^*cap-h *^or MO^*cap-d*2 ^caused phenotypes that were indistinguishable from *cap-g*^*s*105 ^mutants (Figure 5). Retinal patterning in *cap-h *or *cap-d2 *morphants was preserved, although the number of retinal cells within each of the three layers was severely reduced. Moreover, nuclei within *cap-h *or *cap-d2 *morphant retinal cells were of highly irregular shapes and enlarged, similar to the phenotypes observed upon loss of CAP-G. The phenotypic similarities between the different mutants and morphants suggest that a single condensin I complex is present in zebrafish.

**Figure 6:**
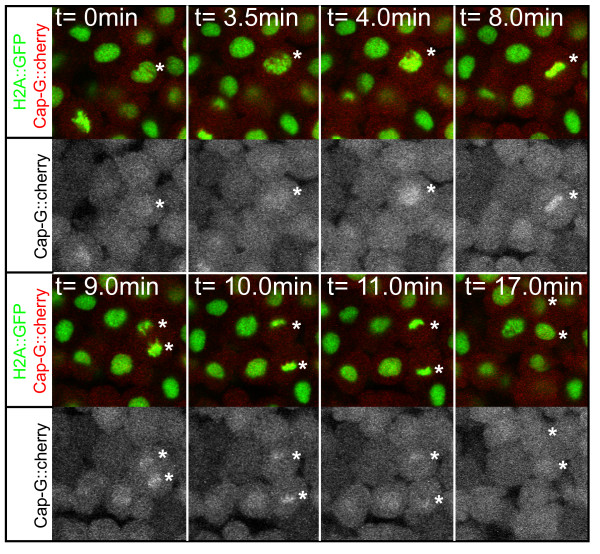
**Chromatid association of CAP-G during mitosis**. Selected images from a timelapse recording of a CAP-G-mcherry fusion protein in a gastrula stage transgenic embryo. The fusion protein associates with chromatids after the breakdown of the nuclear envelope at the beginning of prometaphase (t = 4.0 min) where it remains until decondensation of chromosomes during telophase (t = 17.0 min). Asterisks indicate positions of segregating chromatids. The different mitotic stages are recognizable by transgenic *H2A::GFP *expression. M, metaphase; PM, prometaphase; T, telophase.

**Figure 7:**
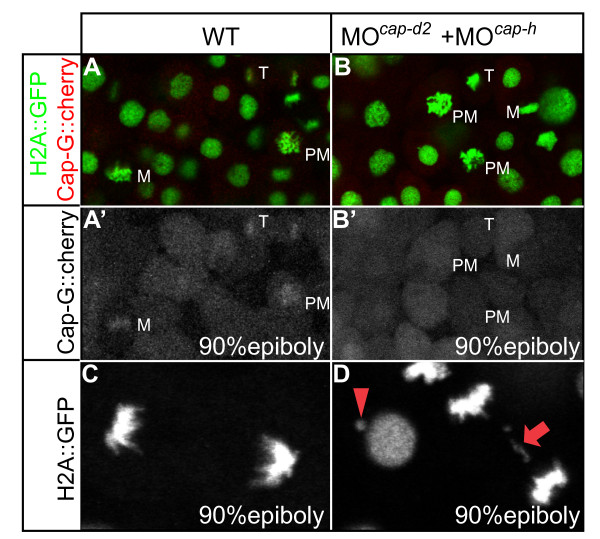
**CAP-G-mcherry is mislocalized during gastrula stage mitoses upon loss of its interaction partners CAP-H and CAP-D2 and localizes to the cytoplasm**. (A, B) The different mitotic stages are recognizable by transgenic *H2A::GFP *expression. M, metaphase; PM, prometaphase; T, telophase. (C, D) Occurrence of chromatid bridges in MO^*cap-h*+*cap-d*2^ injected embryos. Red arrow indicates a chromatid bridge in between two anaphase nuclei. Red arrowhead marks aberrant genetic material associated with a decondensed nucleus.

To test the functional conservation of CAP-D2 and CAP-H within the zebrafish condensin I complex, we generated an expression construct encoding a fusion protein between CAP-G and red-fluorescent cherry and visualized the dynamics of CAP-G localization during mitosis in gastrula stage embryos. In wild-type, CAP-G-mcherry translocated from the cytoplasm into the nucleus at the onset of prometaphase, concurrently with the breakdown of the nuclear envelope. The fusion protein associated with separating chromatids during anaphase and subsequently, during telophase, disappeared from the decondensing chromatids (Figure 6) [see Additional file 6]. Failure to associate with its physical interaction partners CAP-H or CAP-D2 in MO^*cap-h*+*cap-d*2 ^injected embryos resulted in the cytoplasmic mislocalization of the fusion protein throughout the mitotic cycle (Figure 7B). High resolution confocal imaging of mitoses revealed the presence of chromatid bridges in MO^*cap-h*+*cap-d*2 ^injected embryos (Figure 7D). Together, these findings substantiate the functional conservation of CAP-H and CAP-D2 within the zebrafish condensin I complex.

### Loss of the condensin I complex causes polyploidization

To test whether the observed changes in nuclear shapes and sizes could be attributed to polyploidization as a consequence of chromatid segregation defects, we measured the genomic content of wild-type, *cap-g*^*s*105^, *cap-h *morphant, *cap-h*/*p53 *double morphant, or *cap-d2*/*p53 *double morphant retinal cells at 72 hpf by flow cytometry (Figure 4E; Table 2). At this stage, there is little proliferation which could mask the presence of aneuploid and polyploid cells in G_1_/0. This analysis demonstrated a significant and comparable increase in the percentage of retinal cells with a genomic content of 4C (1C = haploid genome equivalent) in all mutant/morphant combinations as compared to wild-type (Table 2). This increase could either be attributed to polyploidy or to G_2_/M phase arrest. As inability of cells to exit G_2_/M typically corresponds to an increase in mitotic nuclei [45], we used the anti-PH3 antibody to label and count mitotic nuclei at 72 hpf in *cap-g*^*s*105 ^mutants. By this analysis, we could confirm that the mitotic index (MI) was low and statistically not significantly different between retinae of both genotypes [wild-type: MI = 0.3% ± 0.2% standard deviation (SD), n = 22/6683 mitotic/total retinal cells; *cap-g*^*s*105^: MI = 0.5% ± 0.5% SD, n = 12/2869 mitotic/total retinal cells; p > 0.05, not significant]. These results argue against G_2_/M arrest in *cap-g*^*s*105 ^mutants and confirm that mitotic events are too few to account for the significant increase of 4C content nuclei. Together, these findings suggest that loss of the condensin I complex results in accumulation of polyploid, and, to a lesser degree, aneuploid cells.

**Table 2:** Accumulation of genomic material in nuclei of *cap-g*^*s*105 ^mutants, *cap-h*, and *cap-d2 *morphants.

	DNA content fraction				
	<2C	2C	2C – 4C	4C	>4C
WT	2.2% ± 0.3%	87.9% ± 0.1%	4.5% ± 0.5%	3.5% ± 0.4%	1.3% ± 0.1%
cap-g^s105^	6.8% ± 1.7%	65.8% ± 4.0%	6.1% ± 0.5%	15.5% ± 1.3%	4.2% ± 0.7%
MO^cap-h^	4.3% ± 1.9%	70.1% ± 3.7%	8.2% ± 0.8%	13.1% ± 0.3%	2.6% ± 1.0%
MO^cap-h+p53^	3.7% ± 1.2%	74.2% ± 8.7%	6.6% ± 2.1%	11.2% ± 3.8%	3.0% ± 1.2%
MO^cap-d2^	9.8% ± 5.4%	61.4% ± 10.7%	11.3% ± 3.0%	10.0% ± 1.5%	4.7% ± 1.5%
MO^cap-d2+p53^	7.6% ± 3.5%	64.4% ± 14.2%	9.3% ± 3.2%	12.2% ± 4.2%	4.8% ± 2.9%

	WT vs. *cap-g*^s105^				
	
	p < 0.05	p < 0.01	p < 0.01	p < 0.001	p < 0.01
	
	WT vs. MO^*cap-h*^				
	
	**p > 0.05**	p < 0.01	p < 0.01	p < 0.001	**p > 0.05**
	
	WT vs. MO^*cap-d*2^				
*t*-test p-values					
	**p > 0.05**	p < 0.05	p < 0.05	p < 0.005	p < 0.05
	
	MO^*cap-h *^vs. MO^*cap-h*+*p*53^				
	
	**p > 0.05**	**p > 0.05**	**p > 0.05**	**p > 0.05**	**p > 0.05**
	
	MO^*cap-d*2 ^vs. MO^*cap-d*2+*p*53^				
	
	**p > 0.05**	**p > 0.05**	**p > 0.05**	**p > 0.05**	**p > 0.05**

Table showing the distribution of nuclear DNA contents among all counted cells. Values represent an average of four independent experiments. *t*-test p-values (*lower table*) reveal a significant shift from diploid to aneuploid and especially tetraploid nuclei in *cap-g*^*s*105^, *cap-h *morphants or *cap-d2 *morphants compared to wild-type. There are no significant differences in the genomic content distributions of *cap-h *morphant and *cap-h*/*p53 *double morphant or of *cap-d2 *morphant and *cap-d2*/*p53 *double morphant retinae.

## Discussion

We have reported the first functional analysis of the condensin I complex in a vertebrate organism. The lack of other homologs of *cap-g*, *cap-h *and *cap-d2 *within the zebrafish genome and phenotypic similarities between the appropriate mutants and morphants suggests that a single condensin I complex is present in zebrafish. High-resolution live imaging of mitoses in *cap-g*^*s*105 ^mutants and *cap-g *morphants revealed that progression through mitosis is delayed and that chromatid segregation defects occur during anaphase. Together these findings confirm a role of the condensin I complex that is conserved between zebrafish and other eukaryotic organisms. Our analysis has extended previous studies from other eukaryotic model systems in demonstrating that the loss of CAP-G, CAP-H or CAP-D2 causes aberrant sizes and shapes of retinal cell nuclei most likely caused by tetraploidization. This is rather a direct effect of chromosomal non-disjunction during anaphase than G_2_/M arrest, as levels of mitotic cells do not increase upon loss of condensin I subunits, as would be expected in case of the latter [45].

In our analysis of the condensin I complex we have focused on retinal development since retinal patterning and proliferation have been well described [38]. The neural retina is a tissue which is largely derived from a small pool of highly proliferative progenitor cells that are located within the CMZ region [41]. We could show that expression of *cap-g*, *cap-h *and *cap-d2 *correlates with the expression of *pcna *which marks regions of cell proliferation within the retina. In comparison, there was no overlap with the expression of *elavl3*, a late stage marker of neurogenesis, which indicates that *cap-g*, *cap-h *and *cap-d2 *expression is down-regulated upon differentiation. We also showed that loss of CAP-G had no effect on the transcriptional expression of *pcna *or *elavl3*, indicating that the severe loss of cell number within the neural retina is not caused by transcriptional silencing of genes involved in proliferation or neurogenesis. Moreover, since several neuronal differentiation markers are correctly expressed, we could not detect any obvious developmental consequences caused by tetraploidization.

Our observation that some mitoses are delayed until their elimination by the apoptotic machinery suggests that this probably is a major cause of death among retinal cells. In a recent study, Plaster and colleagues showed that loss of the DNA polymerase delta catalytic subunit 1 compromises DNA replication, which is followed by apoptosis. Similar to our study, they reported that knockdown of p53 led to a phenotypic rescue of mutants which suggests that p53 eliminates cells that are stalled within the cell cycle but that otherwise can finish their developmental program [46]. Our observation that loss of p53 does not completely prevent the massive reduction of retinal cells in *cap-g*^*s*105 ^mutants indicates that other forms of cell death or a generally slowed proliferation rate affect the retina.

## Conclusion

Our study of the zebrafish *cap-g *mutant has revealed an essential function of the condensin I complex in rapidly proliferating progenitor cells of the retina and for the maintenance of the diploid state of cell types throughout the entire embryo. Our work has extended previous studies performed in invertebrate models or tissue culture systems to the vertebrate organismal level and has enabled us to characterize the effects of polyploidization on differentiation processes within the entire embryo. A recent report has demonstrated that the *Drosophila *Retinoblastoma family protein 1 Rbf1 physically interacts with dCap-D3 and is required for efficient localization of dCap-D3 with chromatin [30]. This interaction has uncovered a potentially important mechanism by which the inactivation of Rbf members contributes to genome instability which is a hallmark of many tumors. Consistent with this finding, mutations in SMC2 and SMC4 subunits were found in several cases of pyothorax-associated lymphoma [47] and loss of heterozygosity at the Cap-D3 locus is frequently associated with breast cancer [48]. As we have now reported similar genomic instability defects in *cap-g *mutants, it is tempting to speculate that the loss of non-SMC condensin I components will find a similar correlate with tumorigenesis.

## Methods

### Fish maintenance and stocks

Zebrafish were maintained at standard conditions. Embryos were staged by hpf at 28.5°C and fixed at desired timepoints using 4% paraformaldehyde (PFA) in PBS. The following fish strains were used: wild type AB, wild-type WIK, *Tg *[*H2A::GFP*] [44]. The *cbl*^*s*105 ^mutation (Tüpfel longfin background) was isolated during an ethyl-nitrosourea mutagenesis screen performed in the laboratory of Herwig Baier [36,37].

### Mapping, cloning and genotyping of *cap-g*^*s*105 ^mutants

To map the *cap-g*^*s*105 ^locus, F2 embryos from a mapping cross between heterozygous carriers of the *cap-g*^*s*105 ^allele (Tüpfel longfin) and wild-type (WIK) homozygotes were genotyped using a panel of 2982 F2 homozygous mutant embryos. The ZFIN gene names of the condensin I complex genes are as follows: *cap-g*, [ZFIN:si:dkeyp-26a9.1]; *cap-h*, [ZFIN:zgc:158618]; *cap-d2*, [ZFIN:si:dkey-175g20.1].

The following markers were utilized for mapping:

25.11_fw: 5'-AACGGAAATGTAAAATGTAAAACTTGAAT-3'

25.11_rev: 5'-AATAGAGGTGTAAATATGGTCTGAAATGT-3'

zK148F2b_fw: 5'-CTTGTCACAAACTTTCTTTCTTGTGT-3'

zK148F2b_rev: 5'-ACGAATTATCTTTGGTGTTATTCTCA-3'

For genotyping of *cap-g*^*s*105 ^mutant embryos, we used the following primer pair:

ZC209P5a_fw: 5'-GAACTGAACTTCCAACTTTACAACAA-3'

ZC209P5a_rev: 5'-TTTGTTACCAAAACCAAAATACAGAA-3'

### Generation of the *cap-g-mcherry *fusion construct

We designed PCR primers to amplify full-length *cap-g *cDNA which was inserted into the pCS2+ vector. Next, we PCR amplified *monomeric cherry *from Gateway construct p3E mcherry poly A and subcloned the insert into the pCS2+ *cap-g *expression vector at the 3' of the coding sequence.

### Injections of mRNAs and antisense oligonucleotide morpholinos

Constructs were transcribed using the SP6 MessageMachine kit (Ambion). For overexpression (to determine the subcellular localization patterns), 75–100 pg of mRNA were used. For injections of the ATG-directed MOs, the following concentrations were used: 200 μM (MO^*cap-g*^; MO^*cap-h*^; MO^*cap-d*2^) and 100 μM (MO^*p*53^)[43].

MO^*cap-g*^: 5'-CAGATCCGCGTCTCCAGGCATGATG-3'

MO^*cap-d*2^: 5'-CGGAACCATAAAATCCCACGACATC-3'

MO^*cap-h*^: 5'-ACTAAATGCGCTCATAACGAAACTG-3'

MO^*p*53^: 5'-GCGCCATTGCTTTGCAAGAATTG-3'

### Antibodies, immunohistochemistry and sections

Antibody stainings were performed as previously described [49]. The following antibodies were used: rabbit anti-activated caspase 3 (1:200, BD Pharmingen), rabbit anti-phosphorylated histone 3 (1:1000, Upstate Biotechnology), zn5 to label ganglion cells (1:1000, Oregon Monoclonal Bank), zpr1 to label red/green double cones (1:200, Oregon Monoclonal Bank). Nuclei were counter-stained with propidium iodide or DAPI (both 1:1000). Phalloidin labeled with Alexa Fluor 647 was used to mark actin (1:100, Molecular Probes). For sectioning, stained embryos were postfixed over night at 4°C in 2%PFA, 0.3 M sucrose. Embryos were embedded in 4% low melting agarose and sectioned on a Leica VT1000 Vibratome. Confocal images were obtained using the Zeiss LSM 510 Meta confocal microscope with a 63× oil lens and zoom 1–3×. Whole embryos were documented under a Leica MZFLIII stereomicroscope using the 1× and 4× objectives with 5–10× zoom and Leica IM50 software package. Photos were processed using Photoshop (Adobe).

### In situ hybridization

Whole mount *in situ *hybridization was carried out as described [50]. Stained embryos were mounted in glycerol and images taken with an Axioplan2 microscope (Zeiss) equipped with a SPOT CCD-camera (Diagnostic Instruments). Templates for probe synthesis were generated from 72 hpf wild-type cDNA by PCR:

CAP-G_fwd: 5'-GCCATTGTCTGGGAGTTTTC-3'

CAP-G_rev: 5'-ATTCTTTGCAGTGGCAGCTT-3'

CAP-H_fwd 5'-GTCATCGACCTTCACGGAGT-3'

CAP-H_rev: 5'-TACGCTGAAACATGGGATCA-3'

CAP-D2_fwd: 5'-TGAAGATGAGCGTGTTCCTG-3'

CAP-D2_rev: 5'-GAAAGCCTTCACACCTGAGC-3'

pcna_fwd: 5'-AGCCACTCCTCTGTCCAAGA-3'

pcna_rev: 5'-AAGGGTTGACTGGATGAACG-3'

elavl3_fwd: 5'-CAAGGCTATCAACACGCTCA-3'

elavl3_rev: 5'-GGGGACAGGTTGTAGACGAA-3'

### DNA content analysis and apoptosis detection

For a single preparation, approx. 50 embryos were collected in E3 medium (5 mM NaCl, 0.17 mM KCl, 0.33 mM CaCl2, 0.33 mM MgSO4), deyolked in 1/2 Fish Ringer without Calcium (55 mM NaCl, 1.8 mM KCl, 1.25 mM NaHCO3), and washed with Hanks. Retinae dissociated from the embryo proper after 20 min incubation with 0.25% trypsine at 4°C. Single cell suspension was achieved after 20–30 min trypsine treatment and nuclei were stained with propidium iodide according to Shepard et al. [51]. Profiles were recorded on a FACS Canto II flow cytometer (Becton Dickinson). Analysis was carried out with CellQuest Pro for n = 4 independent experiments for each condition. Standard deviations and probabilities associated with Student's *t*-test (2-tailed, paired) were calculated using Microsoft Exel software.

The TUNEL assay was performed on cryosections using the "Terminal deoxynucleotidyl transferase mediated dUTP Nick End labelling" and the "*In situ *cell death detection kit, TMR red or Fluorescein" according to the manufacturer's instructions (Roche).

### Time-lapse analysis and live imaging

For time-lapse analysis, embryos were embedded in 1.5% low melting agarose (NuSieve GFT agarose; Cambrex). Spontaneous movements of embryos at 32 hpf were reduced with 3-aminobenzoic acid ethyl ester (Tricaine) (Sigma). *Tg[H2A::GFP] *transgenic embryos were imaged on the Zeiss LSM 510 Meta confocal microscope using 40× magnification and a capture rate of 1 frame per either 30 seconds or 60 seconds. Data were collected and analyzed using Zeiss LSM software. Individual image files were cropped and processed using Photoshop (Adobe) and the movies were assembled using ImageJ software.

### Nuclear circularity and cross section area measurements

Confocal sections of retinae taken at 63× magnification, 0.7× zoom at a resolution of 1024 × 1024 pixels with a Zeiss LSM 510 Meta were first processed with Photoshop software (Adobe) and subsequently imported into Image J software  for further analysis of particle measurements for circularity and area. Area sizes were converted from pixels to μm^2 ^by recalculating measurements taken from the original LSM images. In total, properties of 100–200 nuclei per cell layer and condition were analyzed with Microsoft Excel software. Significance of differences in between nuclei of different conditions was assessed by Student's *t*-test (2-tailed, unpaired, unequal variance). Circularity values for nuclei range between 1.0 (perfectly round) and 0.0 (stretched line).

### Mitotic cell count and Immunohistochemistry with α-Phospho-Histone H3

Analysis was performed according to Shepard et al. [45]. Mitotic cells were counted for only one side of the embryo. Probabilities were calculated in Excel using t-test (2-tailed, unpaired).

## Authors' contributions

SS carried out the positional cloning of the mutation and initiated the functional characterization of the phenotype. FCP carried out the flow cytometry, further characterization of the mutant phenotypes and participated in drafting the manuscript. PG carried out the initial rough mapping of the *cbl* mutation. The mutation was isolated in a screen performed in HB lab. HB also supported the initial mapping effort. WAH supervised the work of PG. SAS carried out the live imaging of mitoses, the characterization of fusion proteins and drafted the manuscript.

## Supplementary Material

Additional file 1 **Conservation of zebrafish Cap-G**. (A) Multiple alignment of vertebrate Cap-G ortholog protein sequences. Conserved regions of identical residues are highlighted. Conserved HEAT domains have been assigned according to the literature [52] and are designated by grey boxes. Zebrafish Cap-G shares 51% identity with human NCAPG overall and 71% identity within the highly conserved N-terminal HEAT repeats (101-279aa). (B) N-J tree representation of phylogenic relationships between eukaryote Cap-G orthologs determined by ClustalW alignment of protein sequences. Accession numbers of sequences used in A and B: *Danio rerio *Cap-G [NCBI:XP_001921367.1]; *Gallus gallus *Cap-G [NCBI:XP_420769.2]; *Homo sapiens *Cap-G [NCBI:NP_071741.2]; *Mus musculus *Cap-G [NCBI:NP_062311.1]; Tetraodon nigroviridis Cap-G [Ensembl:ENSTNIP00000007284]; *Xenopus laevis *XCap-G [NCBI:NP_001081856.1]; *Drosophila melanogaster *Cap-G [NCBI:NP_995827.2]; Saccharomyces cerevisiae Ycg1p [NCBI:NP_010612.2] and *Ciona savignyi *Cap-G [Ensembl:ENSCSAVP00000010600]. Click here for file

Additional file 2 **Genes encoding condensin I complex proteins are expressed within highly proliferative tissues**. Comparison of *cap-g*, *cap-h *and *cap-d2 *expression with that of *pcna *by whole-mount *in situ *hybridization. All genes display overlapping expression patterns throughout early development. Expression at the 512-cell stage indicates a strong maternal contribution. At 24 hpf, condensin I genes are most strongly expressed within brain, retina and spinal cord. Within the retina, expression of condensin I genes is within the CMZ which contains the retinal stem cells whereas expression is absent within postmitotic differentiated retinal cells. Click here for file

Additional file 3 **Early lethality of MO^*cap-g *^injected embryos**. Timelapse movie of wild-type (left side) and *cap-g *morphants (right side) between the 50%-epiboly and 6-somite stages. *cap-g *morphants display a high rate of death as evidenced by rupture of the yolk ball between the tailbud and 4-somite stages. The same phenotype was observed for MO^*cap-g*+*p*53 ^co-injected embryos (not shown). Click here for file

Additional file 4 **Progenitor cell division within the neural tube ventricular zone of 32 hpf wild-type embryo**. Confocal time-lapse movie of *Tg[H2A::GFP] *transgenic embryo tracking cell divisions during a 15 min interval. Click here for file

Additional file 5 **Progenitor cell division within the neural tube ventricular zone of 32 hpf *cap-g*^*s*105 ^mutant embryo**. Confocal time-lapse movie of *Tg[H2A::GFP] *transgenic and *cap-g*^*s*105 ^mutant embryo tracking cell divisions during a 15 min interval. Several nuclei are condensing during prometaphase but do not progress to anaphase stages. Click here for file

Additional file 6 **Chromatid association of CAP-G-mcherry during mitosis**. Confocal time-lapse recording of a 25 min interval in a gastrula stage wild-type embryo expressing CAP-G::cherry (changed to grayscale). The dynamic association of CAP-G with chromatids occurs between prometaphase and telophase. Click here for file
