# Supplementary material for: Non-SMC condensin I complex proteins control chromosome segregation and survival of proliferating cells in the zebrafish neural retina
Source: BMC Dev Biol. 2009 Jul 8;9:40. doi: 10.1186/1471-213X-9-40 (PMC2727499; doi:10.1186/1471-213X-9-40)
Supplement: Additional file 1 — Conservation of zebrafish Cap-G. (A) Multiple alignment of vertebrate Cap-G ortholog protein sequences. Conserved regions of identical residues are highlighted. Conserved HEAT domains have been assigned according to the literature [52] and are designated by grey boxes. Zebrafish Cap-G shares 51% identity with human NCAPG overall and 71% identity within the highly conserved N-terminal HEAT repeats (101-279aa). (B) N-J tree representation of phylogenic relationships between eukaryote Cap-G orthologs determined by ClustalW alignment of protein sequences. Accession numbers of sequences used in A and B: Danio rerio Cap-G [NCBI:XP_001921367.1]; Gallus gallus Cap-G [NCBI:XP_420769.2]; Homo sapiens Cap-G [NCBI:NP_071741.2]; Mus musculus Cap-G [NCBI:NP_062311.1]; Tetraodon nigroviridis Cap-G [Ensembl:ENSTNIP00000007284]; Xenopus laevis XCap-G [NCBI:NP_001081856.1]; Drosophila melanogaster Cap-G [NCBI:NP_995827.2]; Saccharomyces cerevisiae Ycg1p [NCBI:NP_010612.2] and Ciona savignyi Cap-G [Ensembl:ENSCSAVP00000010600]. [file 1471-213X-9-40-S1.pdf]

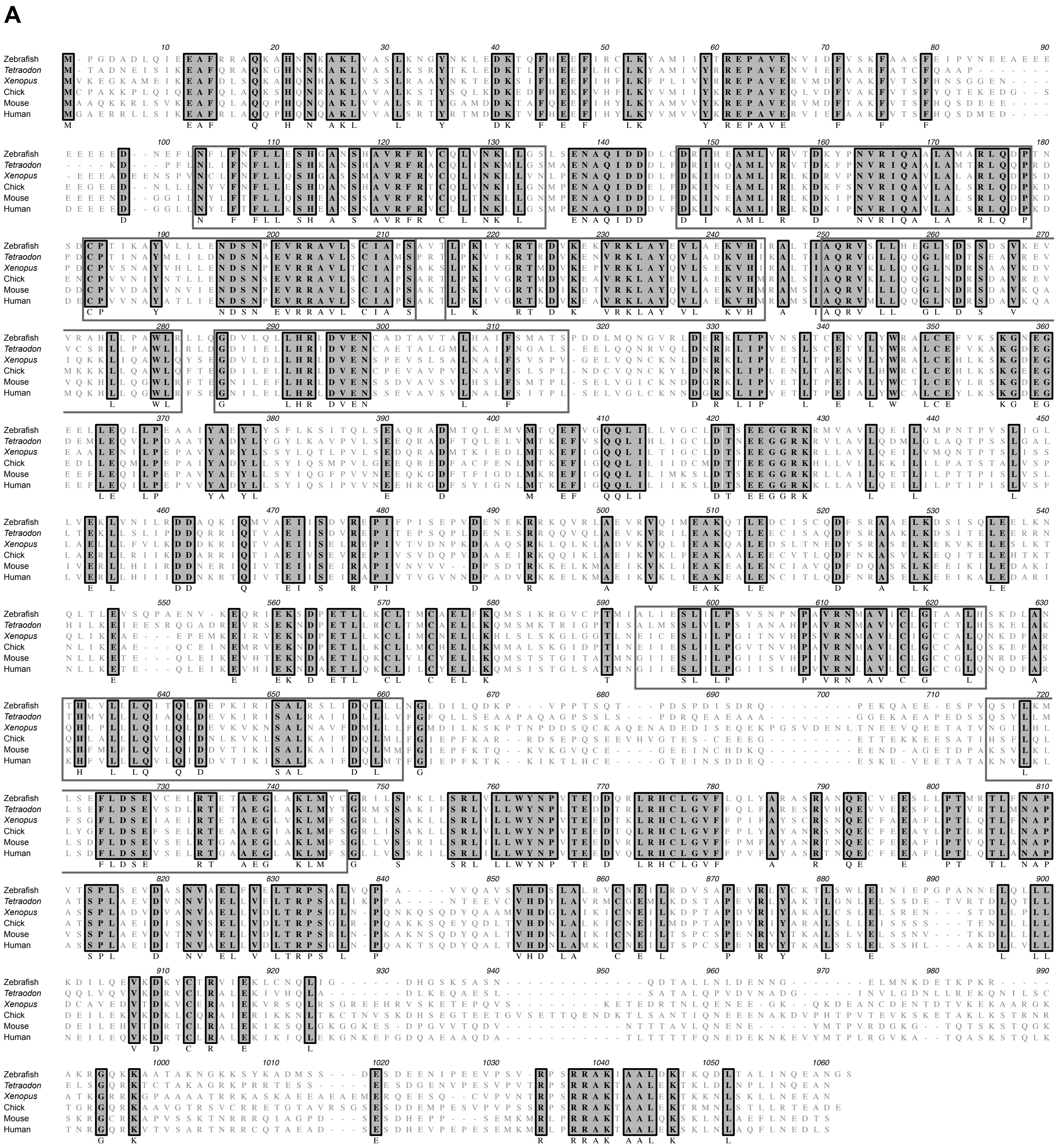
A
Zebrafish
Tetraodon
Xenopus
Chick
Mouse
Human

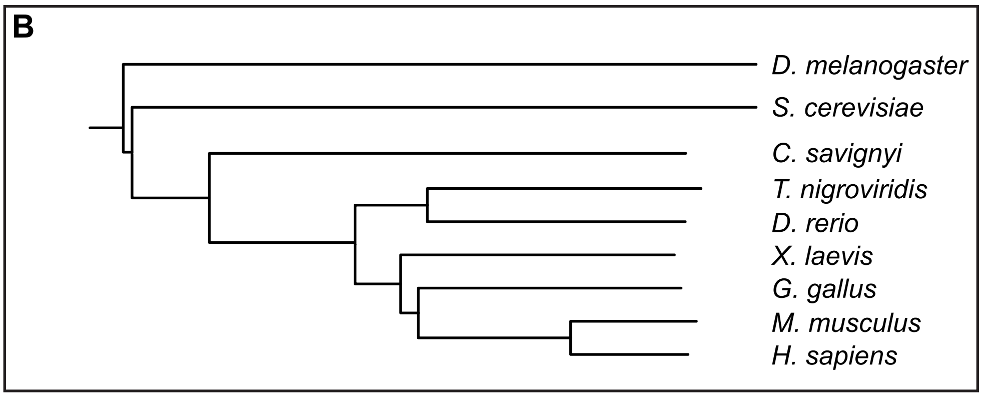
B
D. melanogaster
S. cerevisiae
C. savignyi
T. nigroviridis
D. rerio
X. laevis
G. gallus
M. musculus
H. sapiens
