# Supplementary figures and images for: Non-SMC condensin I complex proteins control chromosome segregation and survival of proliferating cells in the zebrafish neural retina
Source: BMC Dev Biol. 2009 Jul 8;9:40. doi: 10.1186/1471-213X-9-40 (PMC2727499; doi:10.1186/1471-213X-9-40)

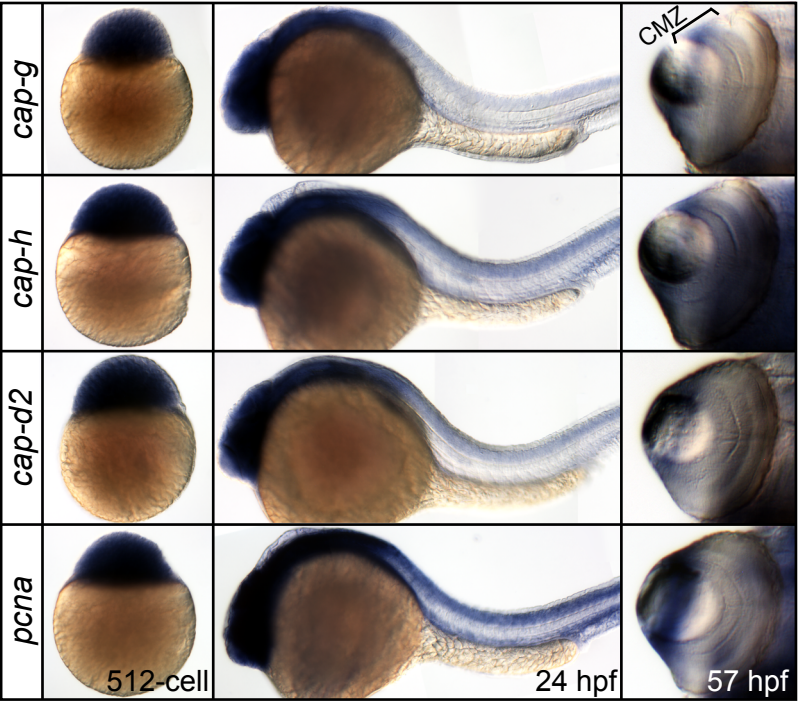

Supplement: Additional file 2 — Genes encoding condensin I complex proteins are expressed within highly proliferative tissues. Comparison of cap-g, cap-h and cap-d2 expression with that of pcna by whole-mount in situ hybridization. All genes display overlapping expression patterns throughout early development. Expression at the 512-cell stage indicates a strong maternal contribution. At 24 hpf, condensin I genes are most strongly expressed within brain, retina and spinal cord. Within the retina, expression of condensin I genes is within the CMZ which contains the retinal stem cells whereas expression is absent within postmitotic differentiated retinal cells. [file 1471-213X-9-40-S2.pdf]
